# Ozone therapy mitigates parthanatos after ischemic stroke

**DOI:** 10.1186/s40659-024-00547-5

**Published:** 2024-10-05

**Authors:** Jiahui Li, Xiaolei Liu, Zengze Wang, Pengyun Xie, Min Zhu, Hanhui Zhong, Sirui Luo, Jing Tang, Guixi Mo

**Affiliations:** The Department of Anesthesiology, Affiliated Hospital of Guangdong Medical University, Zhanjiang, Guangdong China

**Keywords:** Ozone, Parthanatos, ROS, Ca^2+^, Cerebral ischemia–reperfusion

## Abstract

**Background:**

Stroke is a leading cause of death worldwide, with oxidative stress and calcium overload playing significant roles in the pathophysiology of the disease. Ozone, renowned for its potent antioxidant properties, is commonly employed as an adjuvant therapy in clinical settings. Nevertheless, it remains unclear whether ozone therapy on parthanatos in cerebral ischemia–reperfusion injury (CIRI). This study aims to investigate the impact of ozone therapy on reducing parthanatos during CIRI and to elucidate the underlying mechanism.

**Methods:**

Hydrogen peroxide (H_2_O_2_) was utilized to mimic the generation of reactive oxygen species (ROS) in SH-SY5Y cell reperfusion injury in vitro, and an in vivo ischemic stroke model was established. Ozone saline was introduced for co-culture or intravenously administered to mice. Apoptosis and oxidative stress were assessed using flow cytometry and immunofluorescence. Western blotting was utilized to examine the expression of parthanatos signature proteins. The mechanism by which ozone inhibits parthanatos was elucidated through inhibiting PPARg or Nrf2 activity.

**Results:**

The findings demonstrated that ozone mitigated H_2_O_2_-induced parthanatos by either upregulating nuclear factor erythroid 2-related factor 2 (Nrf2) or activating peroxisome proliferator-activated receptorg (PPARg). Furthermore, through the use of calcium chelators and ROS inhibitors, it was discovered that ROS directly induced parthanatos and facilitated intracellular calcium elevation. Notably, a malignant feedback loop between ROS and calcium was identified, further amplifying the induction of parthanatos. Ozone therapy exhibited its efficacy by increasing PPARg activity or enhancing the Nrf2 translation, thereby inhibiting ROS production induced by H_2_O_2_. Concurrently, our study demonstrated that ozone treatment markedly inhibited parthanatos in stroke-afflicted mice. Additionally, ozone therapy demonstrated significant neuroprotective effects on cortical neurons, effectively suppressing parthanatos.

**Conclusions:**

These findings contribute valuable insights into the potential of ozone therapy as a therapeutic strategy for reducing parthanatos during CIRI, highlighting its impact on key molecular pathways associated with oxidative stress and calcium regulation.

**Supplementary Information:**

The online version contains supplementary material available at 10.1186/s40659-024-00547-5.

## Background

Parthanatos is a significant type of programmed cell death in cerebral ischemia–reperfusion injury (CIRI) [1–3], which is dependent on poly (ADP-ribose) polymerases (PARP) activation and poly (ADP-ribose) (PAR) formation [4]. Parthanatos becomes irreversible subsequent to the loss of mitochondrial membrane potential loss, the transfer of apoptosis-inducing factor (AIF) from mitochondria to the nucleus, and the ensuing chromatin degradation [4, 5]. Oxidative stress and calcium overload have been identified as significant contributors to parthanatos [6, 7], which can be induced in various cells by N-methyl-N-nitro-N-nitrosoguanidine (MNNG) [8], hypoxia/ reoxygenation (H/R) [9], acidosis [3] and endotoxin [10] in various cells.

Ischemia/reperfusion (I/R) injury is known to be influenced by reactive oxygen species (ROS) and calcium (Ca^2+^), both of which are the primary contributors to this type of injury. Hydrogen peroxide (H_2_O_2_), the most stable form of ROS, has been reported to generate intracellular Ca^2+^ signals [11]. Given the important of ROS and Ca^2+^ in PARP activation, it is crucial to reduce excessive ROS production and calcium overload is essential to alleviate parthanatos. Some drugs with antioxidant effects, such as quercetin, baicalein, and crocetin [9, 12, 13], have been found to alleviate parthanatos and improve neurological function after ischemic stroke. Additionally, ROS exacerbates neuroinflammation and contributes to cell death by activating inflammatory cells during reperfusion [14].

Ozone, a highly reactive compound, typically induces oxidative stress by generating ROS. However, appropriate ozone administration can elicit antioxidant responses and regulate conditions related to oxidative stress and inflammation [15]. Recent studies using animal models have demonstrated that low-dose ozone preconditioning protects the brain, heart, and kidneys from I/R damage [16–18]. Additionally, the antioxidant activity of ozone is linked to its ability to inhibit ROS production and activate cytoprotective pathways driven by nuclear factor erythroid 2-related factor 2 (Nrf2) and peroxisome proliferator-activated receptor g (PPARg), thus attenuating CIRI [19, 20]. Nevertheless, few studies have explored the effect of ozone on parthanatos both in vivo and in vitro, and the associated mechanisms involved remain unclear.

In this study, we investigated the effect of ozone on parthanatos and the underlying molecular mechanisms in SH-SY5Y cells. We also provide evidence to suggest that ozone may be a potential treatment for ischemic stroke.

## Materials and methods

### Animals

Male C57BL/6 J mice (25 ± 2 g, 8–10 weeks old) were obtained from Guangdong Yaokang Biotechnology Co., Ltd (Guangzhou, China). The animal experiments were carried out in accordance with the experimental animal welfare and security system and were approved by the Animal Ethics Committee of Guangdong Medical University (ethical approval number: GDY2002071).

### Transient middle cerebral artery occlusion (tMCAO) model and drug treatment

The tMCAO model was performed as previously reported [21]. Mice were anesthetized with an intraperitoneal injection of pentobarbital sodium and toluene thiazide. The right common carotid artery, external carotid artery, and internal carotid artery were exposed and separated, and a threaded plug (~ 2 cm) was then carefully inserted from the external carotid artery through the internal carotid artery, reaching into the middle cerebral artery. After 90 min of ischemia, reperfusion was initiated by carefully removing the plug. Mice were randomly divided into the following groups (n = 6): sham, tMCAO, and tMCAO + ozone (tMCAO + O_3_). Ozone saline(20 mg/mL, 1.5 mg/kg) was intravenously administered at the onset of reperfusion. The sham and tMCAO groups were injected with an equal volume of normal saline.

### O_3_ preparation

The O_3_ was produced using ozone treatment apparatus(HUMAZON ProMedic, Germany) and injected into sterile saline immediately after extraction. The O_3_ concentration in ozone sterile saline was measured using a high-precision medical ozone meter (HUMAZON ProMedic, Germany). The concentration range analyzed ranged from 1 to 56 µg/mL, while the gas flow rate varied between 0 and 1,300 mL/min.

### The 2,3,5-triphenyltetrazolium chloride (TTC) staining

The cerebral infarct volume was assessed using TTC staining 24 h after tMCAO. Following anesthesia, the brains of mice were swiftly removed, sliced into 2 mm tissue sections, stained with 2% TTC (Sigma-Aldrich, St. Louis, MO, USA) for 20 min, and then immersed in 4% formaldehyde for 24 h. The infarct area was delineated and analyzed using Image J.

### Neurological scoring

A blinded investigator evaluated the neurological deficit (ND) score 24 h after reperfusion. The scoring system was as follows: (1) no ND (0 points); (2) forelimb weakness and torso turning to the ipsilateral side when held by the tail (1 point); (3) circling to the affected side (2 points); (4) inability to bear weight on the affected side (3 points); and (5) no spontaneous locomotor activity or barrel rolling (4 points).

### Rotarod test

The mice underwent a 2-day training period prior to modeling. Following 24 h of reperfusion, the mice were placed on a suspended rod and the speed was increased from 4 to 40 rpm over 5 min. The trial concluded when the mice fell off the rotarod or after reaching 300 s. Each mouse was tested three times with a 5-min rest period between each test. The average latency was utilized for subsequent calculations.

### Cell counting assay

SH-SY5Y cells (CL-0208, Porcell, Wuhan, China)were cultivated on 96-well plates to perform the cell counting assay. Following treatment with H_2_O_2_ and drugs, a fresh medium containing CCK8 (K009, Zeta Life, CA, USA) was added and incubated at 37 °C for 2 h. An enzyme marker was then used to measure the absorbance at 450 nm.

### Flow cytometry

For apoptosis detection, SH-SY5Y cells were incubated with AnnexinV-PE (559763, BD Biosciences, New Jersey, USA) for 15 min, and 7AAD was added 5 min before detection. To detect ROS, dichloro-dihydro-fluorescein diacetate (DCFH-DA) (S0033S, Beyotime, Shanghai, China) was diluted in a serum-free medium at a final concentration of 10 µM. The cells were incubated in the cell incubator at 37 °C for 20 min. Subsequently,, they were washed three times in serum-free cell culture. For Ca^2+^ detection, cells were removed from the medium, washed with Hank's Balanced Salt Solution (HBSS) solution three times, and cultured at 37 °C for 15 min after adding 1 µM Fluo-3 AM probe (40703ES50, Yeasen, Shanghai, China) working solution. Detection was performed using a FACScalibur cytometer (BD Biosciences, New Jersey, USA), and data processing was done using FlowJo 10.8.1. The fluorescence signal was analyzed on at least 10,000 positive events.

### Western blotting

In western blotting, an SDS-PAGE gel was utilized for electrophoresis. Once the bands reached the desired position, they were transferred to a membrane and blocked with 5% skim milk. A diluted primary antibody was then added and incubated overnight at 4 °C. On the following day, the bands were incubated with secondary antibodies specific to the species. The primary antibodies used are listed in Table 1.

**Table 1:** Primary antibodies used in current study

Primary antibodies	Cat. Numb	Supplier
PARP	9532	Cell Signaling Technology
PAR	AM80	Merck Millipore
AIF	5318	Cell Signaling Technology
p-PPARg	AF3284	Affinity Biosciences
PPARg	16643-1-AP	Proteintech
Nrf2	16396-1-AP	Proteintech
p-NF-kB p65	3033	Cell Signaling Technology
NF-kB p65	8242	Cell Signaling Technology
p-p38	4511	Cell Signaling Technology
p38	8690	Cell Signaling Technology
GAPDH	60004-1-Ig	Proteintech
Lamin B	sc-56144	Santa Cruz

### Measurement of Adenosine triphosphate (ATP) levels

The ATP Assay Kit (S0175, Beyotime, Shanghai, China) was employed to quantify the intracellular ATP levels. Cells were harvested, lysed, and then centrifuged at 4 °C at 12000 g for 5 min, after which the supernatant was collected. Subsequently, 100 µl of ATP working solution was added to the assay well, left at room temperature for 3–5 min, and then measured using a luminometer.

### Cellular nicotinamide adenine dinucleotide (NAD^+^) analysis

The NAD^+^/NADH Assay Kit with WST-8 (S0175, Beyotime, Shanghai, China) was used to assess cellular NAD^+^ levels. Cells were harvested and suspended in 200 µl of NAD^+^/NADH extraction buffer. After centrifugation at 12,000 g for 10 min at 4 ℃, the supernatant was collected. To decompose NAD^+^, 100 µl of the sample was heated in a 60 ℃ water bath for 30 min, and 20 µl of the supernatant was utilized for testing. Alcohol dehydrogenase working solution was added, and the mixture was incubated in the dark at 37 ℃ for 10 min. Optical density at 565 nm was measured after 0 and 15 min using a spectrophotometer.

### Mitochondrial membrane potential (JC-1) assay

The Mitochondrial membrane potential assay kit with JC-1 (C2006, Beyotime, Shanghai, China) was utilized to measure the expression of mitochondrial membrane potential. Cells were incubated with 1 ml of JC-1 staining solution at 37 ℃ for 20 min in the cell incubator. After removing the supernatant, cells were washed twice with JC-1 staining buffer. The mitochondrial membrane potential was observed using a laser confocal microscope (FV10i-DOC, Olympus, Japan).

### Superoxide dismutase (SOD) activity detection

SOD activity was assessed using the total SOD Activity Detection Kit (WST-8 Method) (S0101S, Beyotime, Shanghai, China). The WST-8/enzyme working solution was added as per according to the instructions and then incubated at 37 ℃ for 30 min. The absorbance at 450 nm was measured using a microplate reader.

### Hematoxylin–Eosin (HE) staining

After dewaxing and dehydrating, paraffin sections were stained with Harris hematoxylin for 3–8 min and then differentiated with 1% hydrochloric acid alcohol for several seconds. The sections were subsequently rinsed with tap water and returned to a blue color using 0.6% ammonia. Following rinsing with running water, the eosin dye solution was applied for 1–3 min. The sections were dehydrated, mounted, and examined under a microscope. Images were captured and analyzed.

### Terminal deoxynucleotidyl transferase dUTP nick-end labeling (TUNEL) assay

The neuron apoptosis was detected using the Vazyme Biotech Co., Ltd. (Nanjing, China) kit via TUNEL staining following the provided instructions. Paraffin sections were dewaxed, dehydrated, and subjected to antigen retrieval before adding 50 μl of TUNEL working solution to the sample, which was then incubated in the dark at 37 ℃ for 60 min.

### Immunofluorescence

For SH-SY5Y cells seeded on 12-mm glass coverslips, the process involved fixation with 4% paraformaldehyde and permeabilization with 0.1% TritonX-100 were followed by overnight incubation with the specified primary antibody at 4 ℃, after blocking with 5% bovine serum albumin (BSA) for 30 min at room temperature. Following incubation with fluorescent conjugated secondary antibodies and 4',6-diamidino-2-phenylindole (DAPI), the designated primary antibody was applied to paraffin sections. The sections were incubated in a sealing solution containing 0.5% Triton and 5% BSA and left to incubate at room temperature for 2 h. The fluorescence signal was visualized using an Olympus confocal microscope (FV10i-DOC, Olympus, Japan).

### Transmission electron microscopy

Mice were systemically infused with 4% paraformaldehyde under deep anesthesia, followed by 5 ml of 2.5% glutaraldehyde. The brains of the mice were then extracted, and the cortex was sliced into 1–2 mm small pieces using a blade. The cortex pieces were immersed in 2.5% glutaraldehyde and fixed overnight. Following dehydration, the tissues were embedded in epon and ultra-thin sections were obtained using a microtome. These sections were placed on copper electron microscopy grids and subsequently stained with uranyl acetate and lead citrate. Micrographs were then captured and collected using a transmission electron microscope (Hitachi H7500 TEM, Tokyo, Japan).

### Statistical analysis

Statistical analyses were conducted using GraphPad Prism 9. The Student's t-test was employed to assess differences between two groups. One-way analysis of variance (ANOVA) was utilized to analyze datasets comprising more than two groups with consistent sample sizes, while two-way ANOVA (with Turkey's multiple comparisons test) was used for datasets with varying sample sizes. All graphs depict the mean ± standard deviation (SD). A p-value less than 0.05 was considered statistically significant (* p < 0.05, ** p < 0.01, *** p < 0.001, and **** p < 0.0001).

## Result

### H_2_O_2_ induced parthanatos in SH-SY5Y cells

**Fig. 1:**
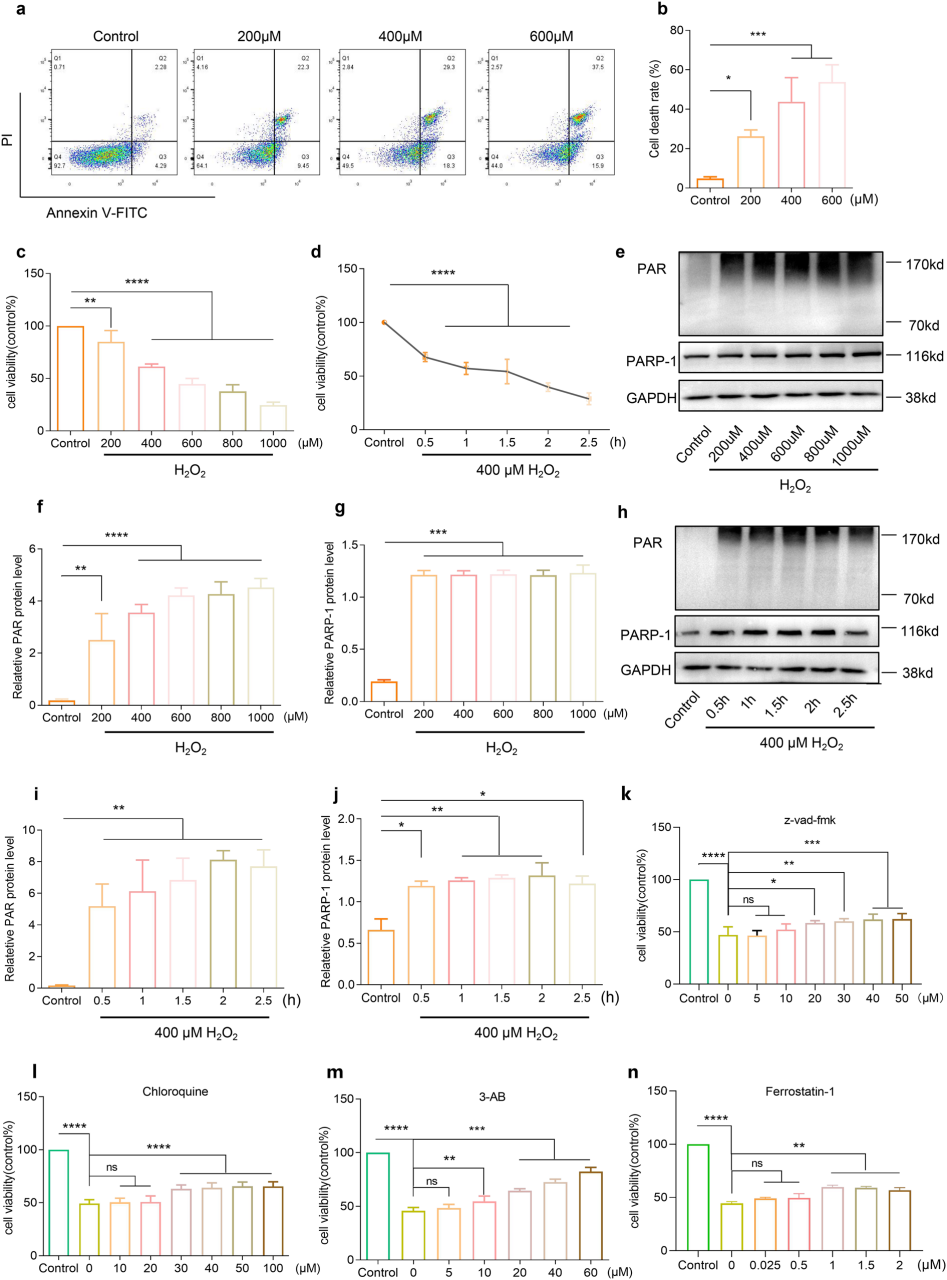
H_2_O_2_ induced SH-SY5Y cells parthanatos. **a** and **b** cell death measurement after different concentrations of H_2_O_2_ by FASC analysis. **c** and **d** measurement of cell viability after different concentrations of H_2_O_2_ and and 400 mM H_2_O_2_ treatment at different time points by CCK8 assay. **e**–**j** PAR protein quantification by WB after treatment with H_2_O_2_ at different concentrations (**e**) and at different time points (**h**). Cell viability measured by CCK8 assay after treatment with z-vad-fmk (**k**), chloroquine (**i**), 3-AB (**m**) or ferrostatin-1 (**n**). Each bar represents the mean ± SD. n = 3, *P < 0.05, **P < 0.01, ***P < 0.001, ****P < 0.0001

Exogenous H_2_O_2_ can initiate PARP1-mediated Parthanatos via DNA damage [22]. Despite this, the precise mechanism underlying H_2_O_2_-induced Parthanatos remains poorly elucidated. In our study, SH-SY5Y cells were exposed to varying doses and durations of H_2_O_2_ to identify the optimal conditions for H_2_O_2_-induced cell death (Fig. 1a–d). Compared to the control group, SH-SY5Y cell viability decreased significantly in a concentration- and time-dependent manner, with 400 mM H_2_O_2_ exerting the significant effect on cell death (Fig. 1e–j). H_2_O_2_ also induced PAR formation and activated PARP-1 within 30 min of treatment. Notably, pretreatment with low doses of z-vad-fmk (a caspase inhibitor), chloroquine (an autophagy inhibitor) or ferrostatin-1 (a ferroptosis inhibitor) demonstrated minimal impact on cellular viability following H_2_O_2_ treatment. Slight increases in cell viability were observed only when the inhibitors reached specific concentrations. In contrast, when SH-SY5Y cells were treated with the same concentration of H_2_O_2_, a significant improvement in cell activity was observed after the use of 3-AB (a parthanatos inhibitor). These results indicate that H_2_O_2_-induced cell death in SH-SY5Y cells involved apoptosis, autophagy or ferroptosis but is primarily dominated by parthanatos (Fig. 1k–n). These findings suggest that H_2_O_2_ predominantly induces parthanatos as a prominent form of cell death.

### Ozone treatment protected SH-SY5Y cells from parthanatos

**Fig. 2:**
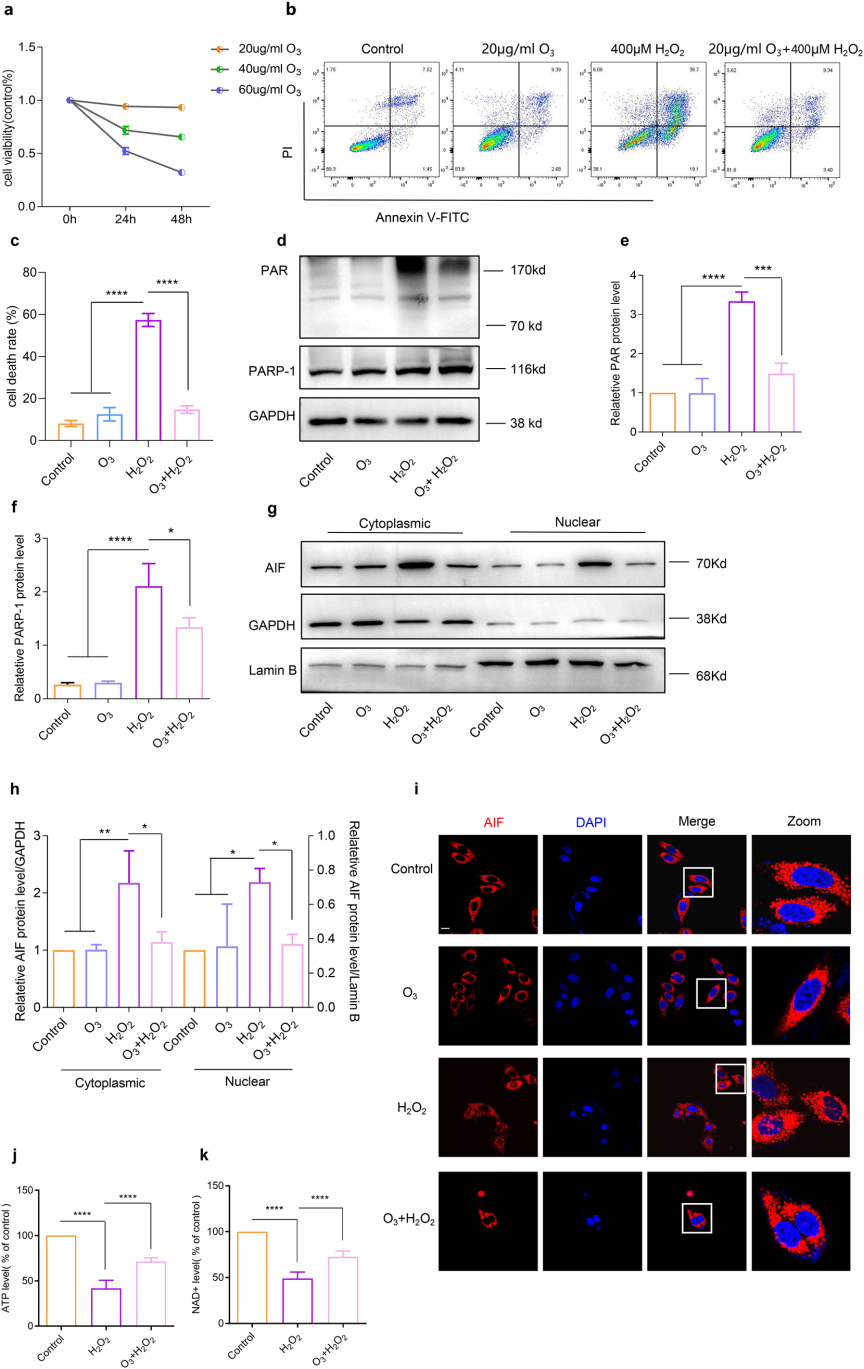
Ozone alleviated H2O2-induced Parthanatos in vitro*.* **a** Cell viability measured by CCK8 assay after treatment with different ozone concentrations. **b** and **c** Cell death measured by flow cytometry. **d**–**i** Quantification of PARP-1 (**d**), PAR (**d**), and AIF (**g**) expression by WB. **i** Immunofluorescence shows the intracellular translocation of AIF (scale bar, 10 mm). **j** Quantification of ATP levels. **k** NAD^+^ activity measured by WST-8. Each bar represents the mean ± SD. n = 3, *P < 0.05, **P < 0.01, ***P < 0.001, ****P < 0.0001

Ozone has been found to exhibit antioxidant properties at high concentrations, while at high levels,it acts act as an oxidant. To investigate the impact of ozone on SH-SY5Y cells, we exposed the cells to varying ozone concentrations for different time intervals. The mean survival rate of SH-SY5Y cells decreased as ozone concentration and incubation time increased, indicating that high ozone concentrations induce SH-SY5Y cell death, whereas low concentrations have minimal effect on cell activity. The identified optimal therapeutic dose of ozone was 20 mg/ml (Fig. 2a).

Subsequent analysis aimed to assess whether ozone could protect SH-SY5Y cells from H_2_O_2_-induced cell death. Treatment with 20 mg/ml of ozone, administered 30 min before exposure to H_2_O_2_, resulted in a significant improvement in the survival rate of SH-SY5Y cells, as evidenced by flow cytometry analysis (Fig. 2b, c). Additionally, we investigated the impact of ozone on parthanatos. Western blot analysis showed that ozone inhibited PARP-1 activation and PAR formation (Fig. 2d–f) and reduced the nuclear level of AIF (Fig. 2g, h). Confocal microscopy also revealed that ozone prevented H_2_O_2_-induced AIF translocation (Fig. 2i). Furthermore, ozone pretreatment alleviated the reduction in ATP and NAD^+^ levels induced by H_2_O_2_ (Fig. 2j, k). These results suggest that ozone protects against H_2_O_2_-induced cell death, particularly involving parthanatos, by inhibiting PARP-1 activation, PAR formation, and AIF translocation to nuclei.

### Ozone prevented parthanatos by decreasing ROS production and Ca^2+^ release

**Fig. 3:**
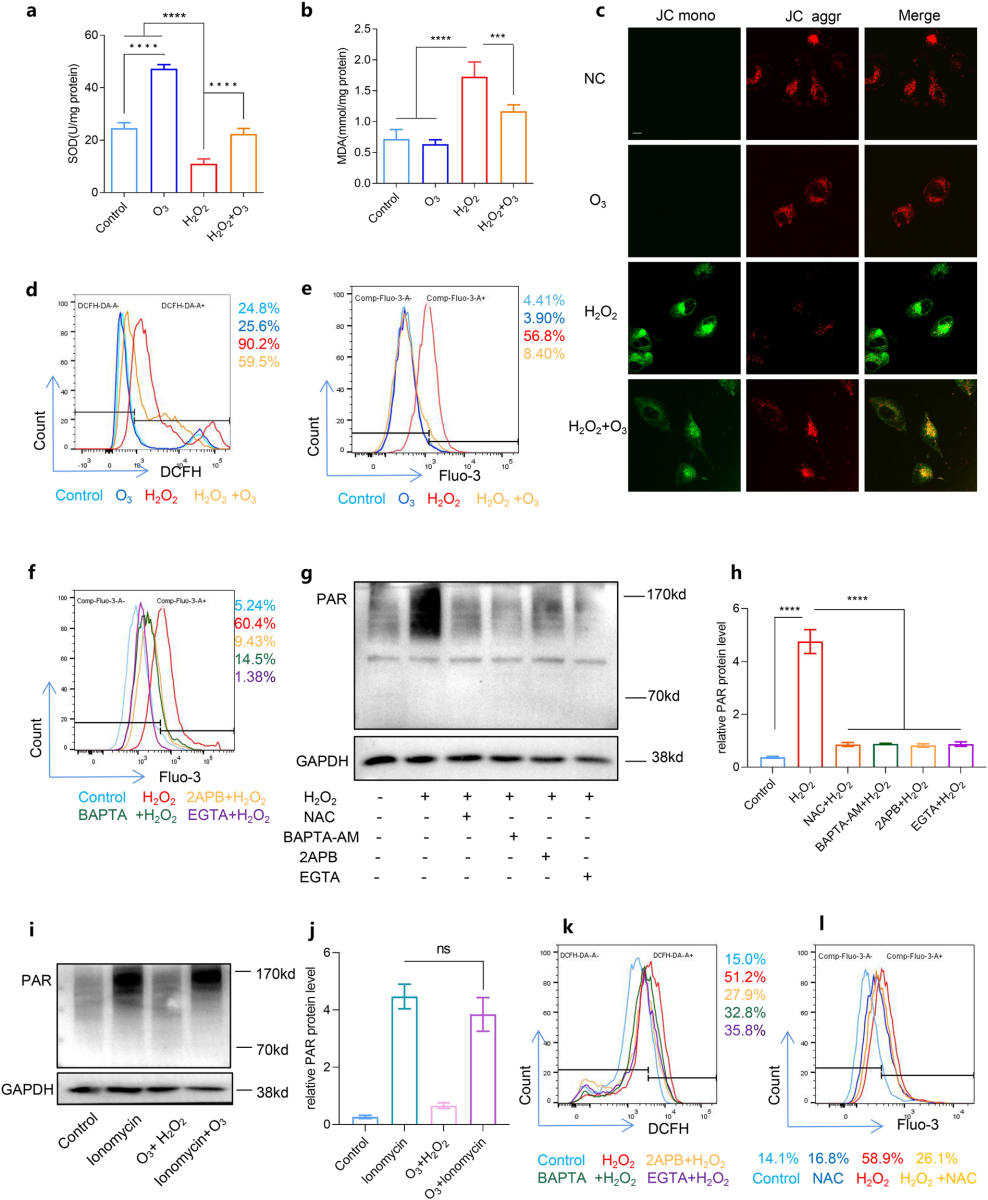
Ozone treatment affected ROS and Ca^2+^ expression after H_2_O_2_ treatment. **a** Total SOD activity measured by WST-8. **b** MDA levels assessed. **c** Mitochondrial membrane potential (JC-1) detected by immunofluorescence (scale bar, 10 mm). Intracellular ROS (**d**) and Ca^2+^ (**e**) expression were measured by flow cytometry after ozone pretreatment. **f** Intracellular Ca^2+^ expression measured by flow cytometry after using various calcium chelators. **g**–**j** Quantification of PAR expression by WB. **k** Intracellular ROS expression after various calcium chelator pretreatment. **l** Intracellular Ca^2+^ expression after NAC pretreatment. Each bar represents the mean ± SD. n = 3, *P < 0.05, **P < 0.01, ***P < 0.001, ****P < 0.0001

We assessed relevant indicators of mitochondrial function due to its association with the occurrence of parthanatos. Following H_2_O_2_ treatment, the activity of the SOD activity was significantly inhibited, and the MDA content increased compared to the control group. However, ozone pretreatment significantly restored SOD activity and reduced MDA levels relative to the H_2_O_2_ alone group (Fig. 3a, b). Additionally, mitochondrial probe JC-1 staining revealed a substantial decrease in green fluorescence (JC monomer) intensity under H_2_O_2_ treatment but a relative increase after ozone pretreatment (Fig. 3c). To determine whether ozone decreases parthanatos by inhibiting ROS production and intracellular Ca^2+^ release, we measured ROS and intracellular calcium levels in SH-SY5Y cells after H_2_O_2_ treatment using the ROS fluorescent probe DCFH2-DA and the cytosolic calcium marker fluo-3 AM, respectively. Ozone pretreatment significantly reversed the H_2_O_2_-induced ROS outburst (Fig. 3d, S1a) and inhibited intracellular Ca^2+^ (Fig. 3e, S1b). Moreover, H_2_O_2_-induced cell death could be partially reversed by the intracellular free-calcium chelator BAPTA-AM, IP3R inhibitor 2APB, or extracellular calcium chelating agent EGTA, which block intracellular calcium, endoplasmic reticulum (ER) calcium release, and extracellular calcium entry, respectively (Fig. S1c). Furthermore, pretreatment with calcium blockers reduced H_2_O_2_-induced cytosolic Ca^2+^ elevation and inhibited PAR generation (Fig. 3g, h). Conversely, PAR formation induced by ionomycin, a Ca^2+^ ionophore, could not be effectively inhibited by ozone treatment (Fig. 3i, j). These results indicate that ozone alleviates H_2_O_2_-induced parthanatos by inhibiting oxidative stress and calcium overload. Moreover, we found that pretreatment with calcium blocker partially reversed H_2_O_2_-induced ROS production (Fig. 3e, S1e), and NAC treatment significantly inhibited intracellular Ca^2+^ compared with the H_2_O_2_-treated group (Fig. 3l, S1f). Furthermore, H_2_O_2_-induced PAR accumulation was mitigated by NAC treatment (Fig. 3g, h). These findings collectively suggest that ROS and Ca^2+^ and their interplay, play a crucial role in parthanatos.

### Ozone alleviated ROS production by upregulating PPARg/Nrf2

**Fig. 4:**
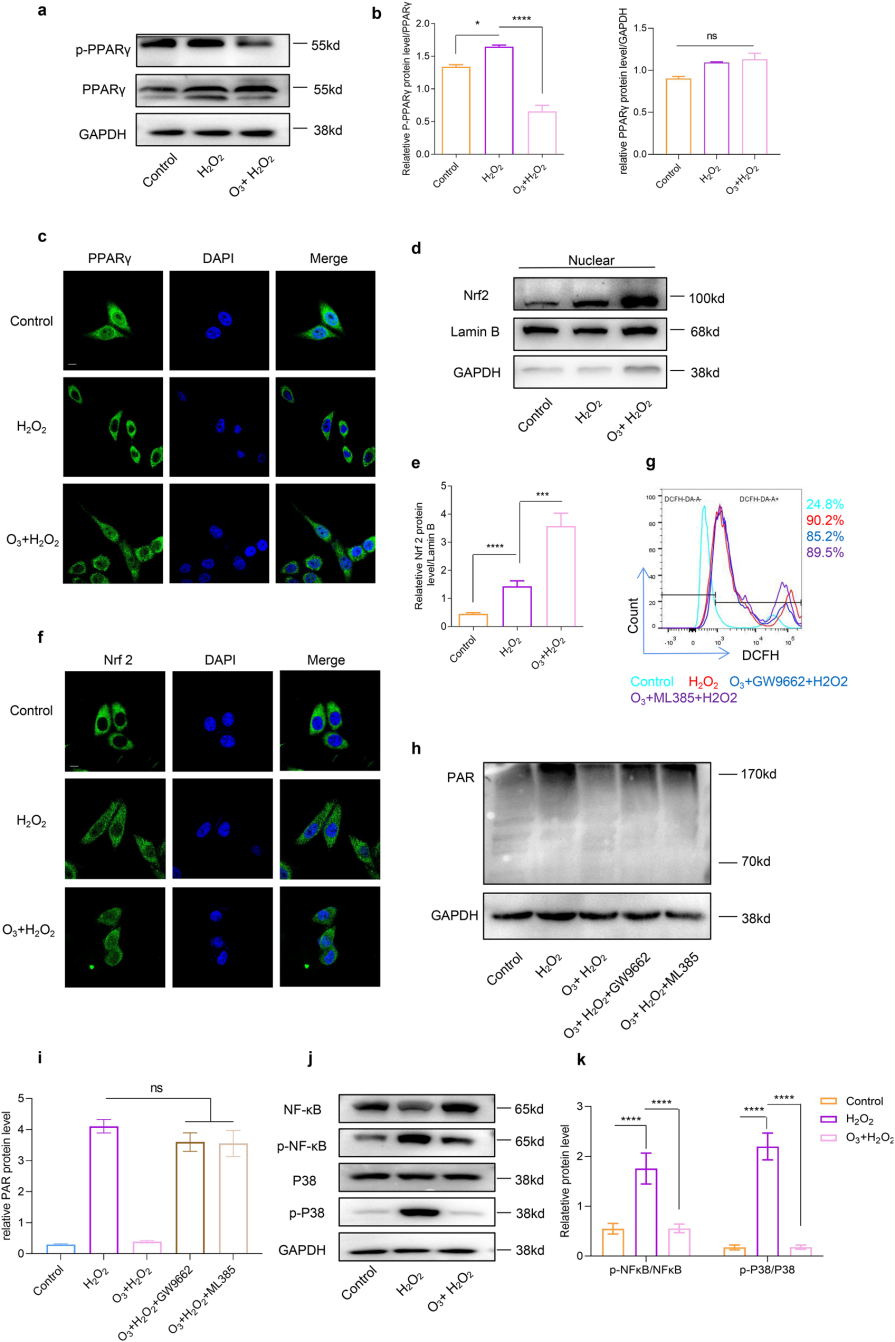
Ozone treatment activated PPARg or Nrf2 to reduce Parthanatos and exert anti-inflammatory effects. **a** and **b**, **d** and **e** Protein expression of p-PPARg, PPARg and Nrf2 in different groups measured by WB. **f** Immunofluorescence showing the intracellular localization of PPARg and Nrf2 after ozone treatment (scale bar, 10 mm). **g** ROS production measured by FACS analysis after PPARg or Nrf2 inhibition. **h** and **i** Protein expression of PAR in different groups measured by WB. **j** and **k** Protein expression of p-NF-kB p65, NF-kB p65 and p-p38/ p38 measured by WB. Each bar represents the mean ± SD. n = 3, *P < 0.05, **P < 0.01, ***P < 0.001, ****P < 0.0001

Upregulation of PPARg/Nrf2 by ozone was observed to alleviate intracellular ROS expression, mitigate mitochondrial damage and regulate intracellular redox balance. Western blot analysis revealed that ozone suppressed the phosphorylation of PPARg, indicating increased nuclear translocation of PPARg in SH-SY5Y cells (Fig. 4a, b). Additionally, ozone treatment significantly upregulated the level of Nrf2 after H_2_O_2_ treatment, and the localization of Nrf2 observed through immunofluorescence was consistent with Western blot results (Fig. 4d–f). Inhibition of PPARg or Nrf2 by GW9662 or ML385 notably increased ROS production, even in the presence of ozone (Fig. 4g, S1g). Moreover, GW9662 or ML385 prevented the reduction of PAR resulting from ozone after H_2_O_2_ treatment (Fig. 4h, i). Ozone was also found to reduce the phosphorylation of P38 and NF-kB p65 induced by H_2_O_2_, indicating its anti-inflammatory effects (Fig. 4j, k). These finding suggest that PPARg or Nrf2 is essential for the antioxidant activity of ozone in parthanatos.

### Ozone protected against cerebral infarction by ameliorating parthanatos

**Fig. 5:**
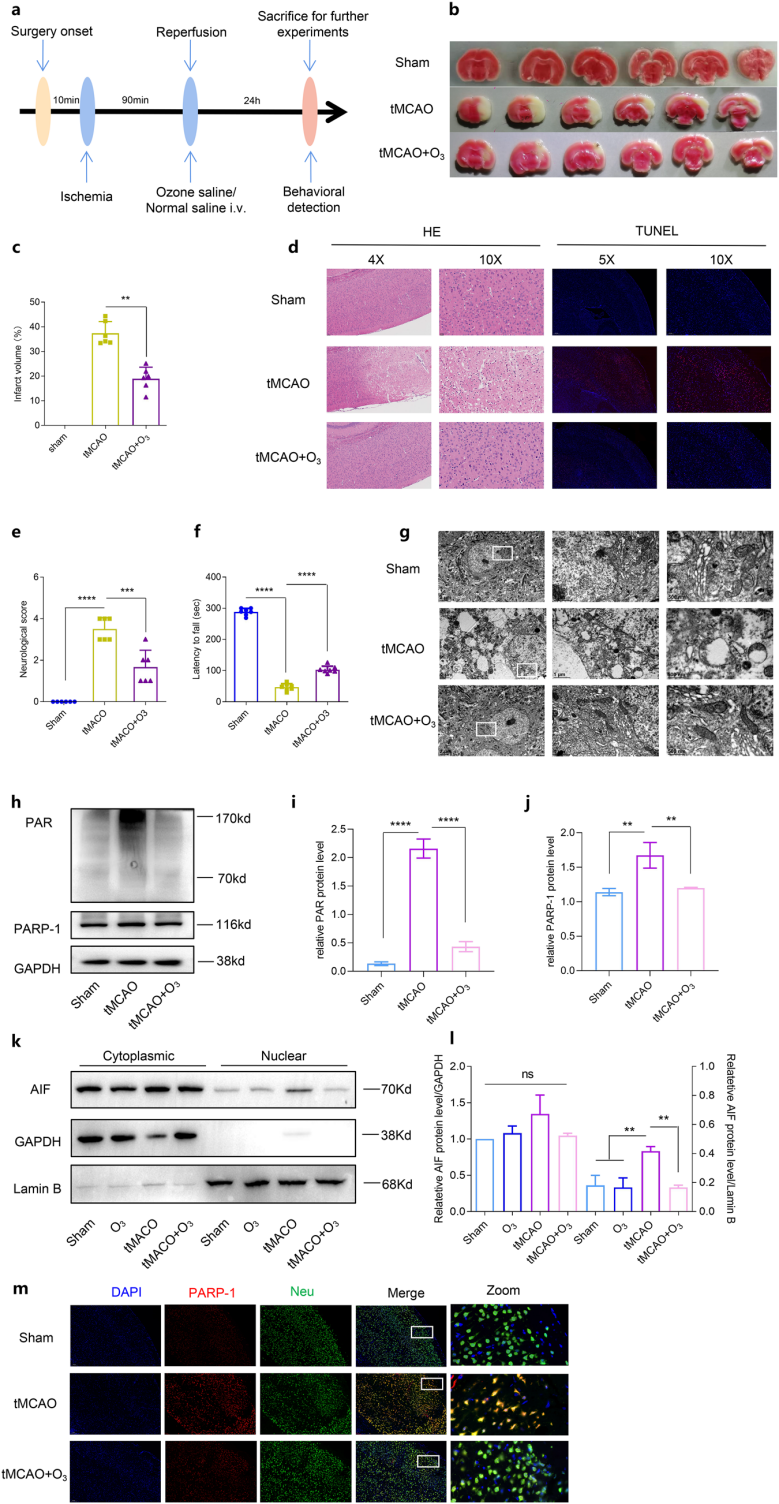
Ozone reduced cerebral infarction and prevents CIRI-induced cortex neuron Parthanatos. **a** Experiment design. **b** and **c** Infarct volume measured by TTC staining of brain slices and percentage. **d** HE and TUNEL staining of brain sections (scale bar, 20 mm). **e** Neurological deficit score of mice. **f** Motor function assessed by rotarod test. **g** Electron micrograph of mitochondria (white) and ER (black) in cerebral cortex neurons. **h**–**l** The protein level of PARP-1, PAR, and AIF measured by WB. **m** Confocal showing PARP-1 expression in cerebral cortex neurons (scale bar, 10 mm). All data shown are the means ± SD. n = 6, *P < 0.05, **P < 0.01, ***P < 0.001

We next investigated the effect of ozone on parthanatos following CIRI in vivo. The experimental design is illustrated in Fig. 5a. TTC staining was employed to assess the infarct size in mice. Ozone treatment significantly reduced the infarct volume compared to the group subjected to CIRI alone (Fig. 5b, c). Furthermore, ozone attenuated the severe neuronal damage observed in the CIRI model group (Fig. 5d). CIRI induced neuronal apoptosis, while ozone treatment reduced apoptosis in the ischemic cerebral cortex (Fig. 5d). Neurological scoring and rotarod tests conducted 24 h after focal I/R demonstrated that ozone-treated mice exhibited improved neurological scores (Fig. 5e) and motor functions (Fig. 5f). These results indicate that ozone protects the brain from I/R injury and enhances neurological function after stroke.

To investigate whether the inhibition of parthanatos induced by stroke is due to the protection of mitochondria and ER by ozone, we employed transmission electron microscopy to observe ultrastructural changes in mitochondria and ER at 24 h after reperfusion. In the CIRI group, mitochondria exhibited swelling, vacuolation, and ridge remodeling, while the ER appeared dispersed and swollen. Ozone treatment improved these damage, resulting in only mild mitochondrial swelling, vacuolar degeneration, reduced ridge debris, and relatively continuous and less swollen ER (Fig. 5g). Western blot analysis revealed that CIRI led to elevated levels of PAR formation, PARP-1 overproduction, and AIF in the nucleus compared to the sham group. However, these outcomes were reversed by ozone therapy (Fig. 5h–l). PARP-1 expression in the cerebral cortex at 24 h after reperfusion was also observed, with consistent results obtained through Western blot analysis (Fig. 5m). Overall, these findings suggest that ozone suppresses parthanatos by mitigating mitochondrial damage and ER during CIRI.

## Discussion

In this study, we delved into the mechanism through which ozone enhances regulation of CIRI by inhibiting parthanatos via the PPARg/Nrf2-ROS-Ca^2+^ signaling pathway. Our findings contribute a novel perspective to understanding the antioxidant capabilities of ozone in the context of ischemic stroke, building upon previously published studies.

Parthanatos stands out as a distinct form of cell death, characterized by its caspase-independent nature, setting it apart from conventional cell death modes like apoptosis and autophagy. Its progression primarily hinges on the rapid activation of PARP-1, synthesis, and accumulation of PAR polymers, mitochondrial depolarization, and nuclear AIF translocation [23–25]. These events culminate in cellular demise, with DNA damage occurring through the ROS-ER Ca^2+^-mitochondria pathway [8]. In this pathway, ROS triggers parthanatos directly, while disruptions in calcium levels prompt mitochondria to release ROS, thereby perpetuating parthanatos. ROS further increases Ca^2+^ release from ER, resulting in a vicious feedback loop [26, 27]. This intricate interplay between ROS, calcium imbalance, and mitochondrial dysfunction underscores the unique and intricate nature of parthanatos as a cell death mechanism.

Ozone therapy, recognized as a natural bioactive molecule with antioxidant properties, has proven successful as an adjuvant or complementary treatment for various diseases [28, 29]. Despite its potential, it's important to note that ozone transforms into an oxidant at high concentrations, contributing to the ongoing controversy surrounding ozone therapy. Several studies have demonstrated the effectiveness of low-dose ozone therapy in treating oxidative stress damage through its antioxidant properties [30]. It has shown efficacy in animal models of diverse conditions, including multiple sclerosis [31], testicular injury [32], experimental sepsis [33, 34] and organic damage [35], memory impairments caused by sleep deprivation [36] and behavioral changes caused by Alzheimer's disease [37]. Ozone plays a regulatory function through Nrf2, inducible nitric oxide synthase (iNOS), NF-kB, and others. Presently, research on ozone therapy is predominantly focused on its antioxidant effects, with limited exploration into its impact on central nervous system diseases. Moreover, few studies have specifically delved into the protective effects of ozone on cell death in CIRI. Notably, recent work by Huang et al. has indicated that ozone protects against I/R-induced brain injury by modulating autophagy in astrocytes [38]. Our study further revealed that ozone influenced parthanatos by regulating Nrf2 and PPARg, subsequently suppressing ROS production and the ensuing calcium release. Since elevated ROS levels cause different types of damage, ozone exhibits significant potential for clinical applications.

PPARg is a ligand-induced nuclear receptor that, when activated and binds to the retinoid X receptor (RXR) to form heterodimers, is transported to the nucleus and neutralizes oxidative stress [39, 40]. In pathological conditions, such as the excessive accumulation of ROS, Nrf2 is translocated into the nucleus, promoting the transcription of protective molecules that defend cells against damage from oxidative stress. Numerous pharmacological experiments have shown that PPARg and Nrf2 play a synergistic role in antioxidant stress and inhibit the NF-kB pathway [41–43]. In the acute injury phase, PPARg directly stimulates the Nrf2/ARE (antioxidant response element) axis to reduce ROS production and restrict tissue damage [40]. Consistently, we observed activation of Nrf2 and PPARg after ozone treatment and inhibition of the NF-kB pathway. This suggests that ozone has both antioxidant and anti-inflammatory effects. Ozone treatment did not control the occurrence of parthanatos when pretreated with PPARg or Nrf2 inhibitor. It may indicate that PPARg and Nrf2 are required for the antioxidant activity of ozone. Although we have proposed that ozone inhibits Parthanatos through PPARg/Nrf2 pathway, the underlying mechanism still requires exploration. Moreover, whether ozone regulates parthanatos through direct interaction with other molecules needs further investigation. In cellular experiments, ozone therapy is currently administered in the form of preconditioning. While it has shown promising results, its ability to replicate clinical efficacy in treating diseases remains uncertain. We cannot definitively assert that post-processing will yield the same outcomes. These aspects necessitate further investigation and comprehensive understanding.

In conclusion, we have demonstrated for the first time that ozone promotes the increased activation of PPARg or Nrf2 translation efficiency, thus inhibiting the production of ROS caused by H_2_O_2_. Additionally, we found that ozone can ameliorate cortical damage in apoplectic mice by antagonizing parthanatos.

## Conclusion

In summary, our findings highlight the significant impact of ozone in inhibiting ROS accumulation after H_2_O_2_ treatment, showcasing its potential in ischemic stroke therapies. We found that ozone attenuated parthanatos by either activating PPARg or upregulating Nrf2 in SH-SY5Ycells. At the same time, we determined that ozone therapy alleviates cortical neuron death in stroke-afflicted mice. These compelling results collectively position ozone as a promising adjunctive treatment strategy for ischemic stroke.

## Supplementary Information

Supplementary material 1

Supplementary material 2

## Data Availability

Not applicable.

## Abbreviations

CIRI: Cerebral ischemia–reperfusion injury
H_2_O_2_: Hydrogen peroxide
ROS: Reactive oxygen species
Nrf2: Nuclear factor erythroid 2-related factor 2
PPARg: Peroxisome proliferator-activated receptorg
PARP: Poly (ADP-ribose) polymerases
PAR: Poly (ADP-ribose)
AIF: Apoptosis-inducing factor
MNNG: N-methyl-N-nitro-N-nitrosoguanidine
H/R: Hypoxia/ reoxygenation
I/R: Ischemia/reperfusion
tMCAO: Transient middle cerebral artery occlusion
TTC: 2,3,5-Triphenyltetrazolium chloride
ND: Neurological deficit
DCFH-DA: Dichloro-dihydro-fluorescein diacetate
HBSS: Hank's Balanced Salt Solution
ATP: Adenosine triphosphate
NAD^+^: Nicotinamide adenine dinucleotide
SOD: Superoxide dismutase
HE: Hematoxylin–Eosin
TUNEL: Terminal deoxynucleotidyl transferase dUTP nick-end labeling
BSA: Bovine serum albumin
DAPI: 4',6-Diamidino-2-phenylindole
iNOS: Inducible nitric oxide synthase
RXR: Retinoid X receptor

## References

[1] Fricker M, Tolkovsky AM, Borutaite V, Coleman M, Brown GC. Neuronal cell death. Physiol Rev. 2018;98:813–80. 29488822 10.1152/physrev.00011.2017 PMC5966715

[2] Tuo QZ, Zhang ST, Lei P. Mechanisms of neuronal cell death in ischemic stroke and their therapeutic implications. Med Res Rev. 2022;42:259–305. 33957000 10.1002/med.21817

[3] Zhang J, et al. Augmentation of poly(ADP-ribose) polymerase-dependent neuronal cell death by acidosis. J Cerebr Blood F Met. 2017;37:1982–93. 10.1177/0271678X16658491 PMC5464694 27381826

[4] Andrabi SA, et al. Poly(ADP-ribose) (PAR) polymer is a death signal. P Natl Acad Sci USA. 2006;103:18308–13. 10.1073/pnas.0606526103 PMC1838747 17116882

[5] Wang Y, Dawson VL, Dawson TM. Poly(ADP-ribose) signals to mitochondrial AIF: a key event in parthanatos. Exp Neurol. 2009;218:193–202. 19332058 10.1016/j.expneurol.2009.03.020 PMC2752872

[6] Mashimo M, Kato J, Moss J. ADP-ribosyl-acceptor hydrolase 3 regulates poly (ADP-ribose) degradation and cell death during oxidative stress. P Natl Acad Sci USA. 2013;110:18964–9. 10.1073/pnas.1312783110 PMC3839768 24191052

[7] Gerace E, et al. PARP-1 activation causes neuronal death in the hippocampal CA1 region by increasing the expression of Ca(2+)-permeable AMPA receptors. Neurobiol Dis. 2014;70:43–52. 24954469 10.1016/j.nbd.2014.05.023

[8] Zhong H, et al. Propofol inhibits parthanatos via ROS-ER-calcium-mitochondria signal pathway in vivo and vitro. Cell Death Dis. 2018;9:932. 30224699 10.1038/s41419-018-0996-9 PMC6141459

[9] Li WH, et al. Baicalein attenuates caspase-independent cells death via inhibiting PARP-1 activation and AIF nuclear translocation in cerebral ischemia/reperfusion rats. Apoptosis. 2020;25:354–69. 32338336 10.1007/s10495-020-01600-w

[10] Xue Q, et al. Erlotinib protests against LPS-induced parthanatos through inhibiting macrophage surface TLR4 expression. Cell Death Discov. 2021;7:181. 34282120 10.1038/s41420-021-00571-4 PMC8290014

[11] Wu L, Li Z, Yao Y. Hydrogen peroxide preconditioning is of dual role in cardiac ischemia/reperfusion. Eur J Pharmacol. 2023;947:175684. 36997049 10.1016/j.ejphar.2023.175684

[12] Riche K, Lenard NR. Quercetin’s effects on glutamate cytotoxicity. Molecules. 2022;27:7620. 36364448 10.3390/molecules27217620 PMC9657878

[13] Wu H, et al. Crocetin antagonizes parthanatos in ischemic stroke via inhibiting NOX2 and preserving mitochondrial hexokinase-I. Cell Death Dis. 2023;14:50. 36681688 10.1038/s41419-023-05581-x PMC9867762

[14] Maccarrone M, Brüne B. Redox regulation in acute and chronic inflammation. Cell Death Differ. 2009;16:1184–6. 19597462 10.1038/cdd.2009.65

[15] Clavo B, et al. Modulation of oxidative stress by ozone therapy in the prevention and treatment of chemotherapy-induced toxicity: review and prospects. Antioxidants. 2019;8:588. 31779159 10.3390/antiox8120588 PMC6943601

[16] Chen H, et al. Ozone oxidative preconditioning inhibits inflammation and apoptosis in a rat model of renal ischemia/reperfusion injury. Eur J Pharmacol. 2008;581:306–14. 18093583 10.1016/j.ejphar.2007.11.050

[17] Zhu L, Ding S, Xu L, Wu Z. Ozone treatment alleviates brain injury in cerebral ischemic rats by inhibiting the NF-κB signaling pathway and autophagy. Cell Cycle. 2022;21:406–15. 34985377 10.1080/15384101.2021.2020961 PMC8855843

[18] Meng W, et al. Ozone protects rat heart against ischemia-reperfusion injury: a role for oxidative preconditioning in attenuating mitochondrial injury. Biomed Pharmacother. 2017;88:1090–7. 28192883 10.1016/j.biopha.2017.01.151

[19] Scassellati C, Galoforo AC, Bonvicini C, Esposito C, Ricevuti G. Ozone: a natural bioactive molecule with antioxidant property as potential new strategy in aging and in neurodegenerative disorders. Ageing Res Rev. 2020;63:101138. 32810649 10.1016/j.arr.2020.101138 PMC7428719

[20] Lacavalla MA, et al. Ozone at low concentration modulates microglial activity in vitro: a multimodal microscopy and biomolecular study. Microsc Res Techniq. 2022;85:3777–92. 10.1002/jemt.24233 PMC9826497 36131631

[21] Liu S, Zhen G, Meloni BP, Campbell K, Winn HR. Rodent stroke model guidelines for preclinical stroke trials (1st edition). J Exp Stroke Transl Med. 2009;2:2–27. 20369026 10.6030/1939-067x-2.2.2 PMC2848489

[22] Yamamoto T, Kida Y, Kuwano K. Mycoplasma pneumoniae protects infected epithelial cells from hydrogen peroxide-induced cell detachment. Cell Microbiol. 2019;21:e13015. 30702185 10.1111/cmi.13015

[23] Andrabi SA, Dawson TM, Dawson VL. Mitochondrial and nuclear cross talk in cell death: parthanatos. Ann NY Acad Sci. 2008;1147:233–41. 19076445 10.1196/annals.1427.014 PMC4454457

[24] Harraz MM, Dawson TM, Dawson VL. Advances in neuronal cell death 2007. Stroke. 2008;39:286–8. 18187674 10.1161/STROKEAHA.107.511857

[25] David KK, Andrabi SA, Dawson TM, Dawson VL. Parthanatos, a messenger of death. Front Biosci-Landmrk. 2009;14:1116–28. 10.2741/3297 PMC4450718 19273119

[26] Zhang F, Xie R, Munoz FM, Lau SS, Monks TJ. PARP-1 hyperactivation and reciprocal elevations in intracellular Ca2+ during ROS-induced nonapoptotic cell death. Toxicol Sci. 2014;140:118–34. 24752504 10.1093/toxsci/kfu073 PMC4081636

[27] Hamanaka RB, Chandel NS. Mitochondrial reactive oxygen species regulate hypoxic signaling. Curr Opin Cell Biol. 2009;21:894–9. 19781926 10.1016/j.ceb.2009.08.005 PMC2787901

[28] Hao K, Tang S, Xie H, Li X, He X. Application of ozone therapy in interventional medicine. J Interv Med. 2019;2:8–11. 34805862 10.1016/j.jimed.2019.05.003 PMC8562154

[29] Elvis AM, Ekta JS. Ozone therapy: a clinical review. J Nat Sci Biol Med. 2011;2:66–70. 22470237 10.4103/0976-9668.82319 PMC3312702

[30] Bocci V, Valacchi G, Corradeschi F, Fanetti G. Studies on the biological effects of ozone: 8. Effects on the total antioxidant status and on interleukin-8 production. Mediat Inflamm. 1998;7:313–7. 10.1080/09629359890820 PMC1781866 9883965

[31] Delgado-Roche L, et al. Medical ozone promotes Nrf2 phosphorylation reducing oxidative stress and pro-inflammatory cytokines in multiple sclerosis patients. Eur J Pharmacol. 2017;811:148–54. 28623000 10.1016/j.ejphar.2017.06.017

[32] Tusat M, Mentese A, Demir S, Alver A, Imamoglu M. Medical ozone therapy reduces oxidative stress and testicular damage in an experimental model of testicular torsion in rats. Int Braz J Urol. 2017;43:1160–6. 28727368 10.1590/S1677-5538.IBJU.2016.0546 PMC5734081

[33] Kapicibaşi HO, Kiraz HA, Demir ET, Adali Y, Elmas S. Pulmonary effects of ozone therapy at different doses combined with antibioticotherapy in experimental sepsis model. Acta Cir Bras. 2020;35:e202000604. 32667585 10.1590/s0102-865020200060000004 PMC7357834

[34] Yamanel L, et al. Ozone therapy and hyperbaric oxygen treatment in lung injury in septic rats. Int J Med Sci. 2011;8:48–55. 21234269 10.7150/ijms.8.48 PMC3020392

[35] Guanche D, et al. Effect of ozone/oxygen mixture on systemic oxidative stress and organic damage. Toxicol Mech Method. 2010;20:25–30. 10.3109/15376510903503107 20017603

[36] Yan YN, et al. Intraperitoneal ozone injection prevents REM sleep deprivation - induced spatial learning and memory deficits by suppressing the expression of Sema3A in the hippocampus in rats. Iran J Basic Med Sci. 2022;25:980–8. 36159327 10.22038/IJBMS.2022.63994.14112 PMC9464347

[37] Lin SY, et al. Ozone inhibits APP/Aβ production and improves cognition in an APP/PS1 transgenic mouse model. Neuroscience. 2019;418:110–21. 31349006 10.1016/j.neuroscience.2019.07.027

[38] Huang N, Gao D, Zhou S, Huang Z. Ozone reduces ischemic damage after a stroke by regulating the autophagy of astrocytes. Ann Transl Med. 2023;11:79. 36819516 10.21037/atm-22-6456 PMC9929848

[39] Villapol S. Roles of peroxisome proliferator-activated receptor gamma on brain and peripheral inflammation. Cell Mol Neurobiol. 2018;38:121–32. 28975471 10.1007/s10571-017-0554-5 PMC5776063

[40] Cai W, et al. Peroxisome proliferator-activated receptor γ (PPARγ): a master gatekeeper in CNS injury and repair. Prog Neurobiol. 2018;163–164:27–58. 10.1016/j.pneurobio.2017.10.002 PMC6037317 29032144

[41] He F, Ru X, Wen T. NRF2, a transcription factor for stress response and beyond. Int J Mol Sci. 2020;21:4777. 32640524 10.3390/ijms21134777 PMC7369905

[42] Abdelhamid AM, Elsheakh AR, Suddek GM, Abdelaziz RR. Telmisartan alleviates alcohol-induced liver injury by activation of PPAR-γ/ Nrf-2 crosstalk in mice. Int Immunopharmacol. 2021;99:107963. 34273638 10.1016/j.intimp.2021.107963

[43] Hsu WH, Lee BH, Pan TM. Monascin attenuates oxidative stress-mediated lung inflammation via peroxisome proliferator-activated receptor-gamma (PPAR-γ) and nuclear factor-erythroid 2 related factor 2 (Nrf-2) modulation. J Agr Food Chem. 2014;62:5337–44. 24865672 10.1021/jf501373a

