# Supplementary material for: Ozone therapy mitigates parthanatos after ischemic stroke
Source: Biol Res. 2024 Oct 5;57:71. doi: 10.1186/s40659-024-00547-5 (PMC11453019; doi:10.1186/s40659-024-00547-5)

Supplement

S1 (A) Fig. 3D histogram of DCFH-DA fluorescence intensity. (B) Fig. 3E histogram of Fluo-3 AM fluorescence intensity. (C) Cell viability of SH-SY5Y cells after using calcium chelators. (D) Fig. 3F histogram of Fluo-3 AM fluorescence intensity. (E) Fig. 3K histogram of DCFH-DA fluorescence intensity. (F) Fig. 3L histogram of Fluo-3 AM fluorescence intensity. (G) Fig. 4G histogram of DCFH-DA fluorescence intensity.

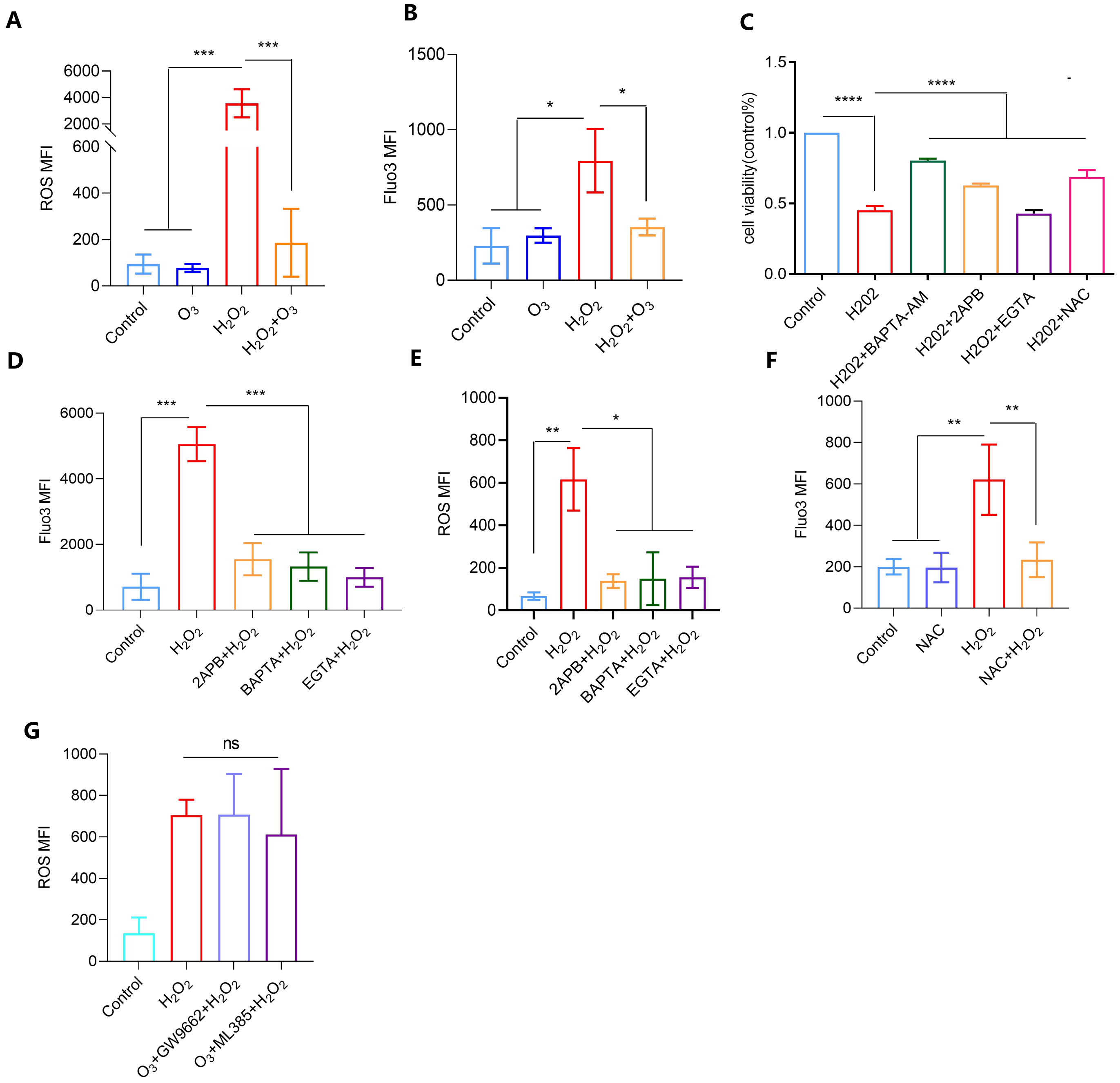

Supplement: Supplementary file 1 — Supplementary material 1 [file 40659_2024_547_MOESM1_ESM.pdf]
