# Supplementary material for: Ozone therapy mitigates parthanatos after ischemic stroke
Source: Biol Res. 2024 Oct 5;57:71. doi: 10.1186/s40659-024-00547-5 (PMC11453019; doi:10.1186/s40659-024-00547-5)
Supplement: Supplementary file 2 — Supplementary material 2 [file 40659_2024_547_MOESM2_ESM.pdf]

1. The transient middle cerebral artery occlusion (tMCAO) model is constructed as described in this article (PMCID: PMC2848489 DOI: 10.6030/1939-067x-2.2.2 RODENT STROKE MODEL GUIDELINES FOR PRECLINICAL STROKE TRIALS (1ST EDITION). J Exp Stroke Transl Med 2, 2-27 (2009).)

In reality, this model is a well-established classic, and we implemented it on mice to elevate the experimental complexity. We made every effort to adhere to your specifications, as detailed below:

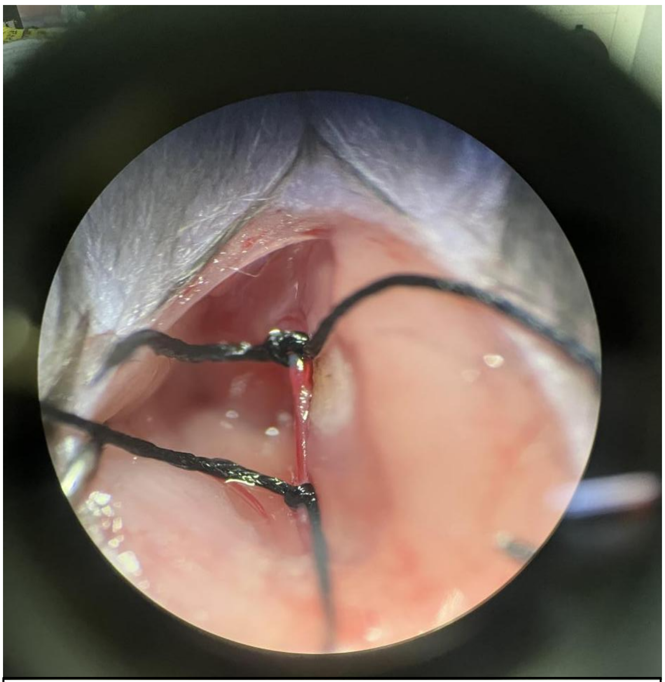

The right common carotid artery, external carotid artery, and internal carotid artery were exposed and separated.

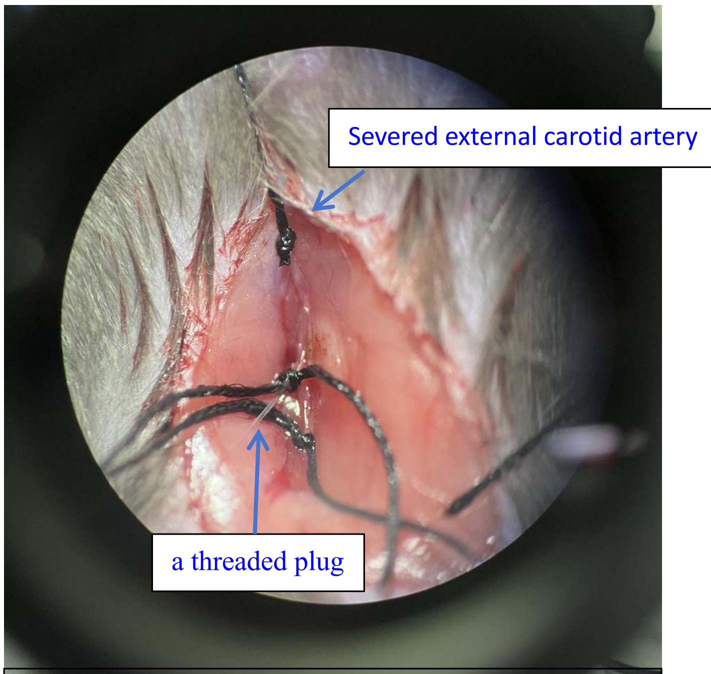

A threaded plug (~2 cm) was then carefully inserted from the external carotid artery through the internal carotid artery, reaching into the middle cerebral artery.

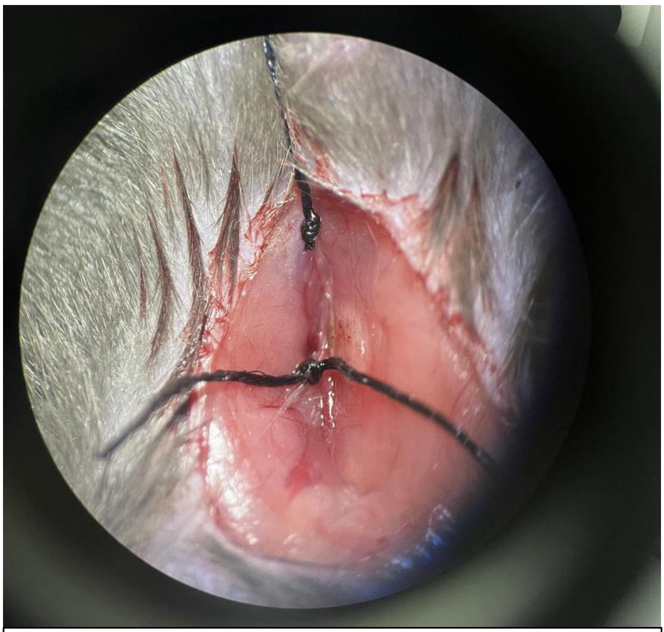

A threaded plug (~2 cm) was then carefully inserted from the external carotid artery through the internal carotid artery, reaching into the middle cerebral artery.

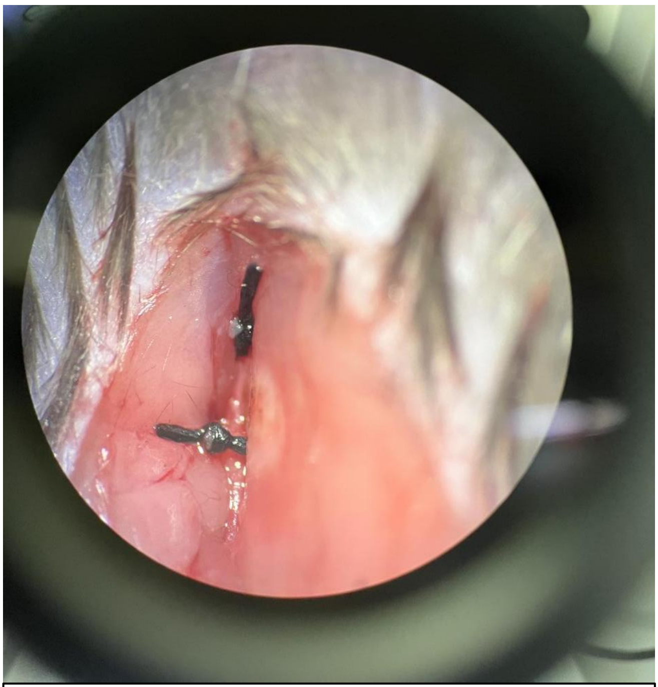

Ozone saline was injected into the tail vein while the plug was pulled out.
